# Money, Mind, and Molars

**Published:** 1911-01-07

**Authors:** 


### MONEY, MIND, AND MOLARS.

### THEIR IMPORTANCE IN DUODENAL ULCER.

The monetary and mental effect which a particular line of treatment may have on a patient is often a factor that escapes the notice of the doctor. By the former we do not mean the expense of a high fee for a consultation or the higher fee for an operation. When once told that such is necessary most patients, if they can afford it, face the necessary expenditure with equanimity. The expense that patients object to is rather the long-continued dribble from the purse resulting from an extended course of treatment, or, more than all, an enforced idleness during which much money is being paid out and none is coming in. This is a point which must be borne in mind in considering the advisability of certain operations. The double convalescence of an appendicectomy in the quiescent period, where the time advocated is six weeks after the attack, in place of the more usual fortnight, is a point that must by no means be lost sight of. Again, a medical course of treatment for gastric ulcer may ultimately be more expensive to the patient than an operation, owing to the longer period of time over which it extends, and this expense may be aggravated by the increased cost of the diet to which the patient is subjected. Many patients, indeed, cannot stand the perpetual dribble of coin and time that is lost in a course for such a disease, and for that reason operation may more confidently be urged early in the less wealthy than the very rich. If a patient has an unlimited supply of money, it may be well to try many forms of treatment before urging operation. If, however, the patient be but of moderate wealth, the physician must feel some qualms in advocating a course of which he is not sure, when a more sure surgical procedure is at hand.

The second effect, the mental, is equally important. Many persons will not persevere with a given form of treatment for a long period of time. Sometimes this is by direct neglect, at others rather by inadvertence, or by sheer inability to keep the mind fixed on one line of treatment. Closely allied to these are the people who cannot follow such a line because the food nauseates them, who cannot bear to be in bed, or who will not take some particular type of food or drug. The whole subject is closely allied to faith-healing. The strong-willed individual who wants and means to get well will stick to the line that his doctor lays down; the less strong-minded cannot help himself, and so cannot get better.

We are glad to see these principles recognised by Dr. Geo. Herschell in his pamphlet on "The Non-Surgical Treatment of Duodenal Ulcer."* In this he has put very clearly the lines along which medical treatment should proceed, and the reasons for these; while, however, he advocates this form of treatment rather than the surgical, he does not lose sight of the fact that the patients may revolt against the monotony of the fare that is necessary. We think this is one of the most important factors of the repeated failures of medical treatment in cases of duodenal ulcer. The other, we think, is the inefficient way in which treatment of the accompanying oral sepsis is carried out. All physicians speak of the need of correcting defects of the teeth, yet how often are patients sent to the surgeon for operation with marked pyorrhœa alveolaris, with many carious and defective teeth. No patient can be thoroughly effectively treated for duodenal ulcer until all pyorrhœa alveolaris has been cured. All bad teeth must be seen to by a dentist. Those are the first points to bear in mind. With this we most heartily agree, but we will go further, and urge that unless those things are done the patients have not been properly treated, either surgically or medically, and that to do a big operation like gastro-enterostomy without attention to these points is bad treatment. We can earnestly recommend Dr. Herschell's little volume, and think it will have done much good if it impresses this last point on the profession.

* "The Non-Surgical Treatment of Duodenal Ulcer." By Dr. George Herschell. London: Henry J. Glaisher. Pp. 39. Price 1s.

